# Rumor detection based on Attention Graph Adversarial Dual Contrast Learning

**DOI:** 10.1371/journal.pone.0290291

**Published:** 2024-04-22

**Authors:** Bing Zhang, Tao Liu, Zunwang Ke, Yanbing Li, Wushour Silamu

**Affiliations:** 1 School of Information Science and Engineering, Xinjiang University, Urumqi, China; 2 Xinjiang Multilingual Information Technology Laboratory, Xinjiang University, Urumqi, China; 3 Xinjiang Multilingual Information Technology Research Center, Xinjiang University, Urumqi, China; Peking University, CHINA

## Abstract

It is becoming harder to tell rumors from non-rumors as social media becomes a key news source, which invites malicious manipulation that could do harm to the public’s health or cause financial loss. When faced with situations when the session structure of comment sections is deliberately disrupted, traditional models do not handle them adequately. In order to do this, we provide a novel rumor detection architecture that combines dual comparison learning, adversarial training, and attention filters. We suggest the attention filter module to achieve the filtering of some dangerous comments as well as the filtering of some useless comments, allowing the nodes to enter the GAT graph neural network with greater structural information. The adversarial training module (ADV) simulates the occurrence of malicious comments through perturbation, giving the comments some defense against malicious comments. It also serves as a hard negative sample to aid double contrast learning (DCL), which aims to learn the differences between various comments, and incorporates the final loss in the form of a loss function to strengthen the model. According to experimental findings, our AGAD (Attention Graph Adversarial Dual Contrast Learning) model outperforms other cutting-edge algorithms on a number of rumor detection tasks. The code is available at https://github.com/icezhangGG/AGAD.git.

## 1 Introduction

With the rapid development of social media, social media has become the main source of people’s daily information acquisition. Compared with previous forms of news media, social media posts have the characteristics of short length and fast dissemination, and at the same time the difficulty of information verification increases. Rumors are a form of misinformation, usually defined as stories or statements with unverified truth value, that can mislead the public and cause significant economic and social disruption [1]. Therefore, false news in social networks has caused great concern, and much effort has been invested in this area to reduce its harm to society, which has been slightly successful but still faces great challenges.

The rise of deep learning has made a very significant contribution to rumor detection, and Li et al. [2] used to automatically and efficiently learn a vector of informative features containing deep semantics from the text, images, and structure of rumors. Ma et al; Wu et al. [3, 4] have demonstrated that recurrent neural networks (RNN) that incorporate long short-term memory (LSTM) and gate recursive units (GRU), along with their diverse variants, are effective in capturing the temporal relationships among individual posts in a rumor propagation chain. Their findings suggest that these models are suitable for modeling complex sequences of events over time. Lu et al; Yu et al.[5, 6] showed that convolutional neural network (CNN) methods have the ability to learn local spatial feature representations. However, these methods mainly focus on the textual information of rumors and do not pay attention to the structural information of rumor propagation. Therefore, further research on the structural information of rumor propagation is needed. Therefore, in order to be closer to the reality, the graph neural network (GNN) approach has become the focus of attention. (Bian et al.; Lu et al.; NGUYEN et al.; Yuan et al.) The effectiveness of structural information for rumor detection was demonstrated by incorporating propagation structure information into the rumor detection model with good results [5, 7–9].

In spite of the ability of GCN to capture the global information and effectively characterize the features of nodes and edges, it has the limitation of being inadequate in assigning diverse weights to distinct neighbors, thereby limiting its ability to effectively capture the relevance of spatial information. However, in GAT [10], due to the attention mechanism, not only the edge information but also the weights can be used. GAT makes the fusion of node features and graph topology makes the information more effective and successfully solves the problem of insufficient ability of GCN to obtain edge weight information. However, it is found that both GAT and GCN filter node features based on the original adjacency matrix used as input. The original adjacency matrix may be noisy and not optimal, which will limit the effectiveness of the filtering operation. We propose a filtering module based on an attention mechanism to deal with which noise for this kind of problem. For example, in a conversation thread, a root node represents the article and the rest of the nodes represent the comment messages. Comments messages with possible malicious attack comments, grammatical errors, garbled characters and run-on comments can be negative value features in the model.

Contemporary organized malicious comment attacks have become very common in the social media domain, which severely affects the judgment of model detection. In order to learn such hostile malicious comments, we utilize adversarial training to simulate this malicious attack phenomenon and train it as a learning object.

When we simulated the malicious attack problem, we encountered a new problem afterwards. Although it provided us with more learnable features, it did not achieve our desired results. We were inspired by the comparative learning in the image domain. We also found related articles in the field of rumor detection, but the model has some problems in the use of contrast learning, his is used to be relatively low and does not really play the real role of contrast learning in the model. In order to make the contrast learning play a real role in the model. We start from the perspective of getting more effective features. We propose a new dual contrast learning for NLP, a framework that specializes in constructing positive and negative paired data, and contrast learning designed to learn generalized feature representations at different granularities. Specifically, inter-instance contrast learning is used in conjunction with a hard sample selection strategy to facilitate task-relevant discriminative feature learning by specifically constructing instance pairs. Contrast learning is performed in learning features with structural information by contrasting the output of the graph neural network, pulling features that are semantically similar and away from features with a relatively large similarity angle. To increase the negative sample representation capability, we added adversarial training as negative samples in addition to using Dropout. These adversarial features will serve as hard negative examples in contrastive learning, aiding the model in its improvement of feature learning from such arduous specimens. We define the dual contrast learning. The relationship between different training samples is learned by swapping the anchors and targets. Moreover, it is commonly held that the aforementioned adversarial attributes may be perceived as distinct types of perturbations that can be deciphered through intuitive means.

Since the graph neural network has more features after convolution, the direct output will put pressure on the fully connected layer, and it is easy to overfit and affect the model robustness. In order to make the model more stable, we added a feature filtering module to finally help the model to complete the output over and improve the performance.

Our contribution is as follows:

We provide an attention mechanism-based filtering module that addresses the graph neural network noise issue and tries to remove noise such intentionally fabricated hostile information, grammatical errors, and misspelled remarks. As a result, it cleans up the data’s low-value information.

To replicate harmful attack comment information and lessen the effect of malicious comments on the model, we use an adversarial training module. Additionally, it offers more views on learning for the future comparison learning.

For NLP, we create a novel hierarchical double comparison learning approach. To help the adversarial training module produce hard negative data, we apply dropout. Through interactive learning of the anchor and target sites, the double contrast is achieved. To support the contrast learning model, the negative samples used in contrast learning are given increased interference power.

Our experimental results demonstrate the superior performance of our model compared to the current state-of-the-art baseline on authentic datasets.

The rest of the paper is structured as follows. Part II describes the related work. Part III describes the problem definition and model structure details. Part IV describes the model experiments.

## 2 Related work

An important branch of rumor detection technology NLP is important for practical applications in several fields such as social stability and social media purification. Our paper summarizes related work in four areas: rumor detection; graph neural networks; adversarial training; and contrast learning.

### 2.1 Rumor detection

Early rumor detection techniques were mainly devoted to supervised algorithms, which typically combine engineering features from post content, user profiles, and information propagation patterns [11–17]. Allport’s propagation theory analyzes lexical content and information propagation [1].

In recent years, automatic feature extraction by neural network models has become a mainstay of rumor detection research. Ma et al. [3] employ recurrent neural networks (RNNs) to obtain temporal and textual embeddings of source posts and user responses. This approach proves to be more effective than utilizing manually-crafted features. Yu et al. [6] use a convolutional neural network (CNN) approach to learn the high-level interactions between key features scattered in the input sequence that shape important features. Shu et al. [18] not only considered the textual information of the rumor writing style but also included the user’s social activities as auxiliary information in order to enhance the identification of rumor detection. In addition, Volkova et al. [10] used LSTM and CNN structures to extract text features for prediction. Chen et al. [19] combined attention mechanisms and RNNs to focus on different features of text. Liu et al. [20] combined RNN and CNN to obtain user features based on time series. Ma et al. constructed a recurrent neural network with tree structure to capture hidden features from top-down or bottom-up propagation structure and text content. To detect rumors early, Zhou et al. [21] use reinforcement learning to dynamically determine how many responses are needed to classify rumors. Stance and user information also become the subject of research. By using stance prediction as an auxiliary task for multi-task learning, (Li et al.; Wei et al.; Kumar and Carley et al.) [2, 22, 23] have shown that stance prediction plays a crucial role in rumor detection. Lu et al. [5] developed propagation networks based on retweet sequences of users with associated user profiles. This approach effectively captures the correlation between user propagation and the source content of their posts. Since text-only rumor detection no longer meets the needs of today’s structured data, our work focuses on the relevance of structural information between original posts and comments, and how to exploit and take advantage of the structural information.

### 2.2 Graph neural networks

Graph Neural Networks (GNN), Gori et al. [24] learn new feature vectors of nodes by recursive neighborhood aggregation schemes. In recent times, there has been a surge in the adoption of generalized convolutional methods for processing graph-based data. Joan Bruna et al. [25] extended convolution to general graphs via a new Fourier transform of graphs. Kipf et al. [26] proposed the Graph Convolutional Network (GCN). Hamilton et al. [27] introduced GraphSAGE, which generates embeddings by aggregating features directly from the local neighborhoods of nodes. GAT (Graph Attention Network) [28] first introduced the attention mechanism into graphs by learning the importance of neighbors through graphs and aggregating neighbors to learn the representation of nodes in the graph. However, the above graph neural network was proposed for homogeneous graphs. (Ren et al; Wang et al; Bi et al) [29–31] considered the attention mechanism in heterogeneous graph learning through the HAN model, which can effectively learn information from connections defined by multiple meta-paths. The meta-path as a handcrafted feature limits HAN. Furthermore, The Heterogeneous Network (HAN) approach exclusively emphasizes the distinction between various modes of connections that exist among target nodes, mediated by meta-paths, without considering the influence of node content that may be associated with different types of nodes. Bian et al. [7] suggest the utilization of a bipartite graph network for modeling upward and downward propagation structures of information in user comments, with the aim of distinguishing between true and false rumors. Ni et al. [32] use source text and dual attention mechanism of user propagation structure for fake news detection task by graph attention network using dual attention mechanism of source text and user propagation structure. Tian et al. [33];Jia et al. [34] used pre-trained BERT+GAT to simulate the event comment interaction network and also the comment views. Influenced by previous work, the graph composed of source text and commentary conversation threads makes the classification of rumors more informative. Our work focused on GAT and improved it. Also to purify the nodes in the comment section, we explored a filter based on an attention mechanism. Noise filtering before entering the graph neural network is implemented to improve the generalization ability of the model while enhancing the prototype representation.

### 2.3 Adversarial training

Adversarial training was initially used more in CV field, in recent years people have started to apply it in NLP. Goodfellow et al. [35] proposed FGSM, they let the amount of change be exactly in the same direction as the gradient, then our error function will increase, then it will have the maximum change on the classification result. They then improved the FGSM by replacing the sign function to take max normalization on the angle, and FGM used L2 parametric normalization. Theoretically L2 normalization preserves the direction of the gradient more strictly, but max normalization is not necessarily the same as the direction of the original gradient. Aleksander Madry et al. [36] proposed a stronger PGD attack for adversarial training, and FGM directly calculated the adversarial perturbation at once through the epsilon parameter, which may not be optimal to obtain. Therefore PGD was improved by iterating more times to slowly find the optimal perturbation. And it is shown in the paper that adversarial training can lead to a robust model. Shafahi et al. [37] proposed FreeAT to optimize the training speed on the basis of PGD and reduce the resource consumption. Malicious comments have been organized to run rampant in social software. Jiang et al. [38] propose SMART. use smooth induced adversarial regularity to improve model robustness while optimizing the proximity algorithm to avoid catastrophic forgetting. Li et al. [39] propose subspace adversarial training (Sub-AT) which constrains AT (adversarial training) in a carefully extracted subspace. it successfully solves both overfitting problems and greatly improves Robustness.

By learning from adversarial training, we use the inspiration of adversarial training to simulate the attacker’s features using adversarial training to obtain a more discriminative metric space.

### 2.4 Contrast learning

Contrast learning has been a great success in the image domain, it has been successfully applied in many tasks and people have started to use it in NLP. unsupervised contrast learning for evaluating summary quality was proposed by Wu et al. [40]. conducted a study on dialogue generation utilizing contrast learning, where the model is trained to effectively distinguish between positive and negative discourse through a carefully curated selection process. During the process of contrast learning, the goal is to train the dialogue model to assign higher conditional probabilities to positive samples and lower conditional probabilities to negative samples in comparison to the reference model. This methodology is employed to ensure a more accurate and efficient comparison between the two models. (Qiu et al.; You et al.; Zhu et al.) [41–43] Contrast learning rapidly unfolds its application on graph-structured data, which provides better generalization, transferability, and robustness to graph data. Yan et al. [44] use self-supervised contrast learning improved the poor performance on the BERT semantic text similarity task. Sun et al. [45] first tried self-supervised contrast learning in the field of rumor detection.

Inspired by the previous contrast learning, we propose a double contrast learning based on layers. The two contrast learning reduces the textual differences of the same text under different masks while increasing the diversity of negative samples. At the same time, we use the interactive contrast between anchor and target to provide more contrast perspectives for the model. The experimental results prove our idea.

## 3 Methodology

**Fig 1:**
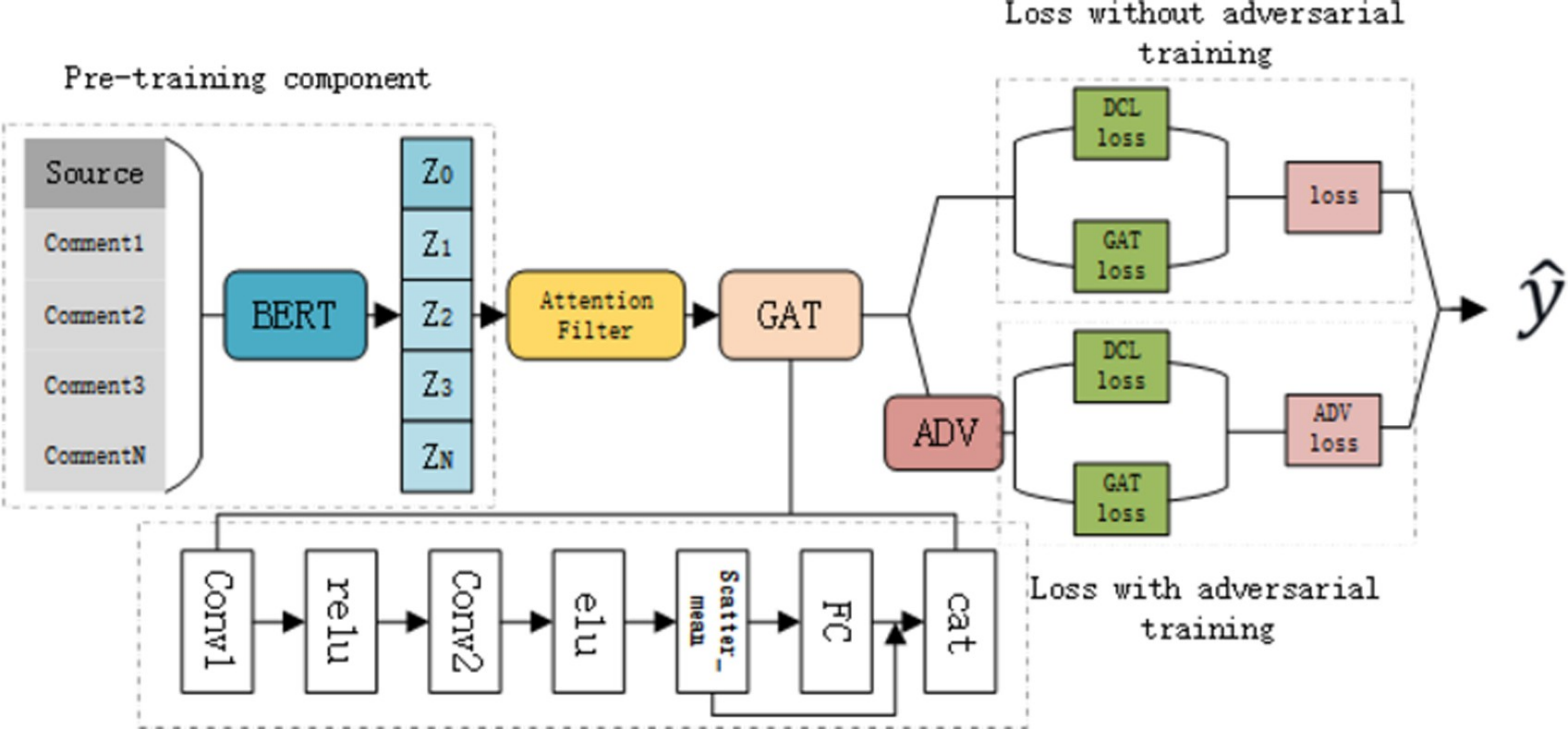
Overview of rumor detection framework for adversarial dual contrast learning graph neural networks.

In this section, we propose an AGAD method in the rumor detection task. Fig 1 illustrates the general framework of our model. Our model can be divided into four parts. Filtering module, graph neural network, adversarial training and contrast learning. Next we will elaborate on the process of classifying rumors by AGAD. Our dataset comes from the work of Sun et al [45].

### 3.1 Task definition

This paper is a rumor detection and classification task. Assume a session thread consisting of source posts and corresponding replies. Let X = {*x*_0_, *x*_1_, · · ·, *x*_*i*_, · · ·, *x*_*N*_} denote a conversation thread, where x0 represents the source post and ${\left\{xi\right\}}_{i=1}^{N}$ represents the N responses. A graph G = (V, E) representing the propagation structure is constructed by treating each element in X as a node and the interactions between the elements as edge connections. V denotes the set of graph nodes and E denotes the set of edges. For example, if nodes *x*_*u*_ and *x*_*v*_ have a direct interaction in the same conversation thread, an edge (*x*_*u*_, *x*_*v*_)∈E is constructed accordingly. Due to the nature of social media, graph G is an acyclic tree. Let y∈{rumor, non-rumor} be the class label. The purpose of rumor detection is to predict a given graph G. In some cases, rumor detection is defined as a four-category task, accordingly, y ∈ {N,F,T,U} (i.e.: Non-rumor, False rumor, True rumor, Unverified rumor).

### 3.2 Attention filter

Our first step is the data processing phase. First, given an input data G, an augmentation operation is performed on the data to obtain the relevant view *G*_*k*_, which contains some subset of the information in the original sample. The corresponding adjacency matrix A of G converts to $A_{k}^{\prime }$ when the probability of de-edging, adding edges or misplacing each training element r. For the text information of graph nodes, dropout mask operation is used to generate text samples with noise containing a small amount of missing information, and BERT [46], which has been successfully used in classification and translation, is used to encode the source code and annotations separately to form a new feature matrix *X*_*k*_. In order to highlight the significance of the source post, a structural linkage is established between the primary post and the associated comment, denoted by [CLS]source[SEP]comment[SEP]. The ultimate hidden state representation of the [CLS] token is utilized to represent the corresponding node representation.

**Fig 2:**
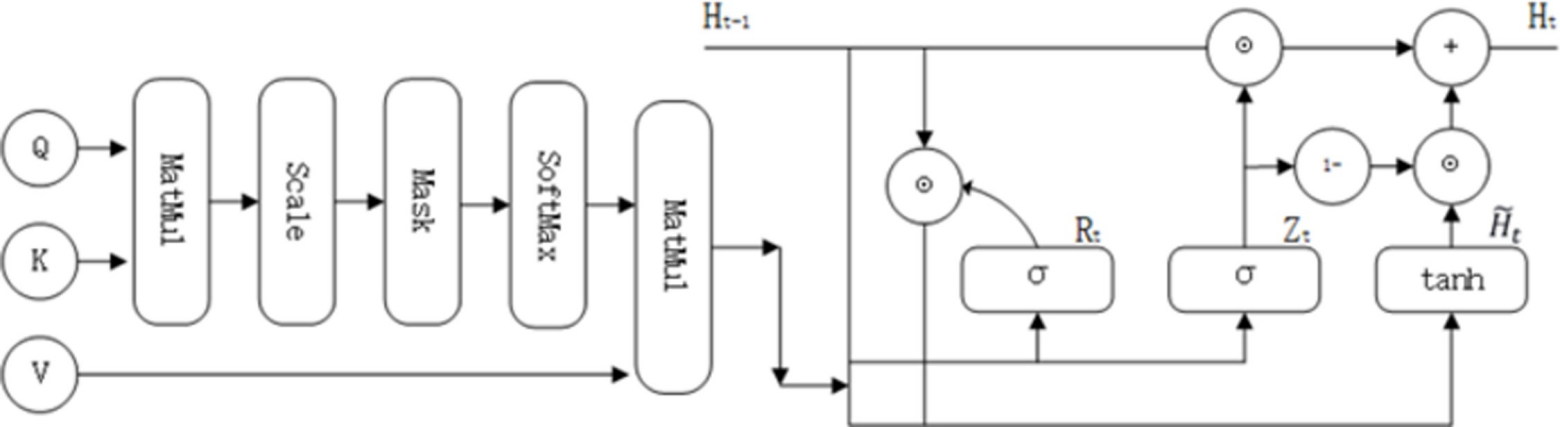
Attention filter structure diagram.

Before input to the graph neural network, we need to filter the incoming data. Although graph neural networks such as GAT and GCN have shown good performance, filtering node features based on the original adjacency matrix used as input. The initial adjacency matrix may exhibit noise and sub-optimal properties, which can hinder the efficiency of the filtering process. To be able to give the model the ability to automatically detect noise, we propose an attention-based filtering module. As shown in Fig 2, we have chosen scaled dot-product attention, which has the following scoring function:

$$\text{a}(q,k)=\frac{q^{T}k}{\sqrt{d}}$$ (1)

Where the dot product operation requires that the query and the key have the same length d. Assuming that all elements of the query and key are independent random variables and all satisfy zero mean and unit variance, then the dot product of the two vectors has mean 0 and variance of d. To ensure that the variance of the dot product remains 1 regardless of the vector length, we divide the dot product by $\sqrt{d}$.

In practice, we usually consider efficiency from a small batch perspective, computing attention based on n queries and m key pairs, where the length of queries and keys is d and the length of values is V. (Query)Q ∈ *R*^*n*×*d*^、(Key)K∈ *R*^*m*×*d*^ and (Value)V∈ *R*^*m*×*v*^ scaled dot-product attention as

$$softmax\left(\frac{QK^{T}}{\sqrt{d}}\right)V\in R^{n\times v}$$ (2)

Upon acquisition of the data pertaining to the enhancement of the weighted attention, it undergoes processing through the filtering module GRU (Gate Recurrent Unit), an iteration-based neural network classified under the class of recurrent neural networks (RNN). We can use the weights to determine the importance and retention of information. The noise with low weights is effectively controlled. The following is the formula for the filter:

$$R_{t}=\sigma \left(X_{t}W_{xr}+H_{t-1}W_{hr}+b_{r}\right)$$ (3)

$$Z_{t}=\sigma \left(X_{t}W_{xz}+H_{t-1}W_{hz}+b_{z}\right)$$ (4)

$${\tilde{H}}_{t}=\tanh\left(X_{t}W_{xh}+\left(R_{t}⊙H_{t-}\right)W_{hh}+b_{h}\right)$$ (5)

$$H_{t}=Z_{t}⊙H_{t-1}+\left(1-Z_{t}\right)⊙{\tilde{H}}_{t}$$ (6)

They are: hidden state *H*_*t*_, candidate hidden state ${\tilde{H}}_{t}$, update gate *Z*_*t*_, and reset gate *R*_*t*_.

The main working principle is: The vector at moment t and the hidden state at moment t-1 are obtained by adding bias to the corresponding weights to obtain *Z*_*t*_, and *R*_*t*_. for the pair use. Then the candidate hidden state *R*_*t*_. is obtained by *R*_*t*_. Finally, the candidate hidden state is determined by updating the gate *Z*_*t*_ to finally obtain *H*_*t*_.

### 3.3 GAT

The currently popular graph convolutional networks have demonstrated excellent capabilities in graph information aggregation. We improved the graph attention network GAT [28], which, unlike the graph convolutional network GCN [26], learns node encoding through iterative multi-headed attention with neighboring nodes and has the advantage of inferring encoding to new nodes after training. To calculate $h_{i}^{l+1}$, the encoding of node i at iteration l+1 is:

$$e_{ij}^{l}=LR\left(a^{l^{r}}\left(W^{l}h_{i}^{l}⊕W^{l}h_{j}^{l}\right)\right)$$ (7)

$$h_{i}^{l+1}=\sigma \left(\sum_{j\in N(i)}softmax\left(e_{ij}^{l}\right)z_{j}^{l}\right)$$ (8)

where LR is the activation function we use a mixture of ReLU and eLU activation functions, ⊕ the concatenation operation, N(i) is the neighbors of node i, $e_{ij}^{l}$ is the non-normalized attention score between nodes i and j, a and w are the scientific system parameters, and $h_{i}^{0}$ denotes the encoding generated by BERT.

Ultimately, mean-pooling operators (referred to as MEAN) are employed as a means of consolidating the wealth of information contained within $h_{i}^{l+i}$ - which represents the node representations within a given set ‐ into a single, cohesive representation. It is formulated as

$$h_{k}=MEAN\left(h_{i}^{l+1}\right)$$

### 3.4 Adversarial training module

**Fig 3:**
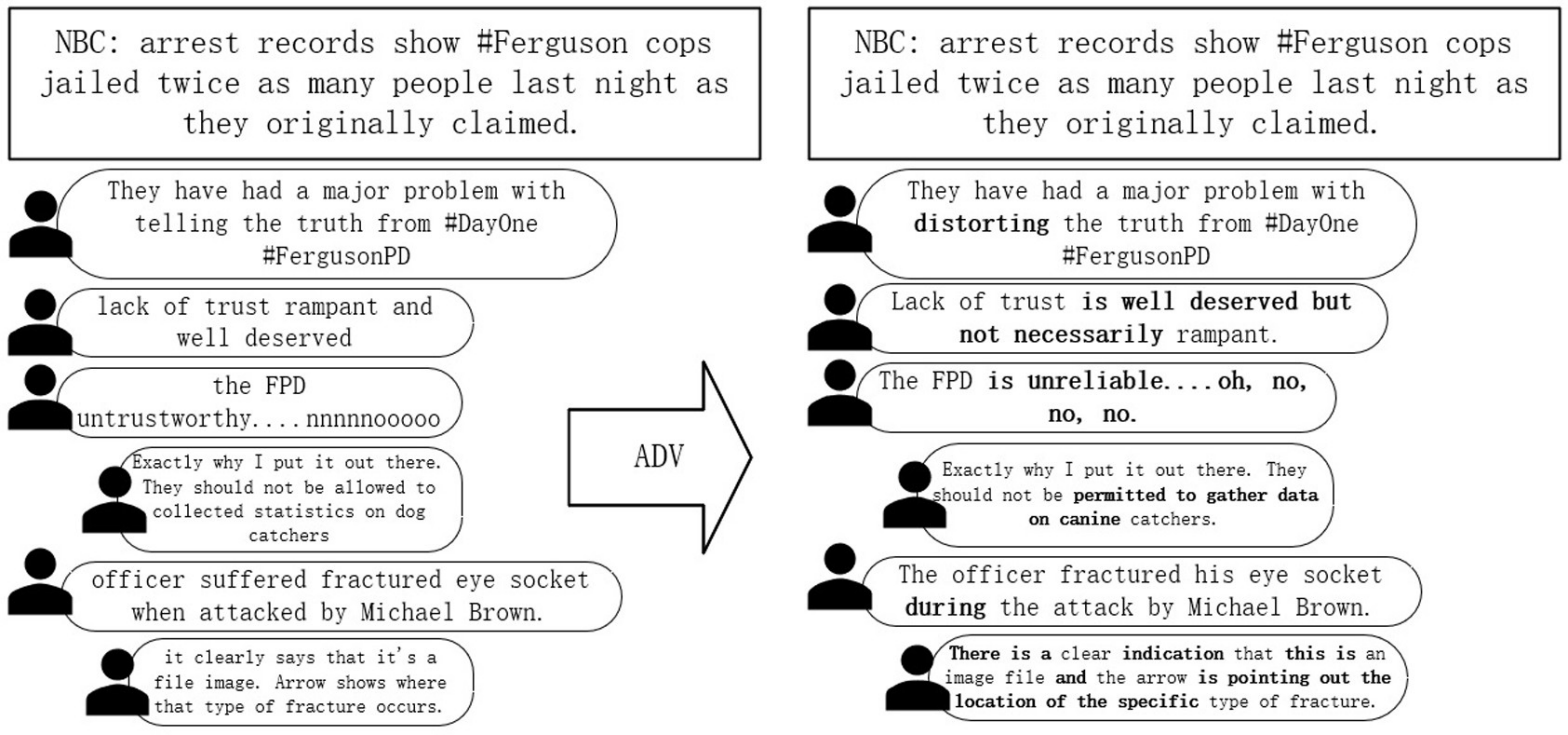
Adversarial training.

We propose an adversarial training module applied to NLP as shown in Fig 3. Rumor mongers may use various camouflage strategies is the session threads are more like the real information, thus making the model performance worse. Ma et al. [47] first proposed a GAN-based adversarial generative network, which eventually generated many adversarial comments to cover up the facts by adversarial training. What we propose is not a generative confrontation, but a perturbed approach to confrontation training. It can be understood as generating adversarial vectors in a high-dimensional space for compressing the model, thus mining time-invariant feature information in the training phase.

We propose adversarial training by repeating the training m times in succession for each sample x, computing r by reusing the gradient of the previous time, and the overall epoch is divided by m to ensure speed.

For each min-batch of samples K gradients are obtained, and each time the gradient is obtained, we use it to update both the perturbation and the parameters. If the inner layer does K iterations of the chemistry, our module divides the overall iteration epoch by K, which ensures that the overall gradient is computed the same number of times as the normal training. From the perspective of outer training, each min-batch is trained the same number of times as normal training, except that the order in which it is trained changes somewhat, with K identical min-batches being trained sequentially. Although successive identical mini-batches do not bring as much perturbation to parameter updates as random mini-batches, experiments show that it does not affect our final convergence effect.

### 3.5 Double comparison learning

**Fig 4:**
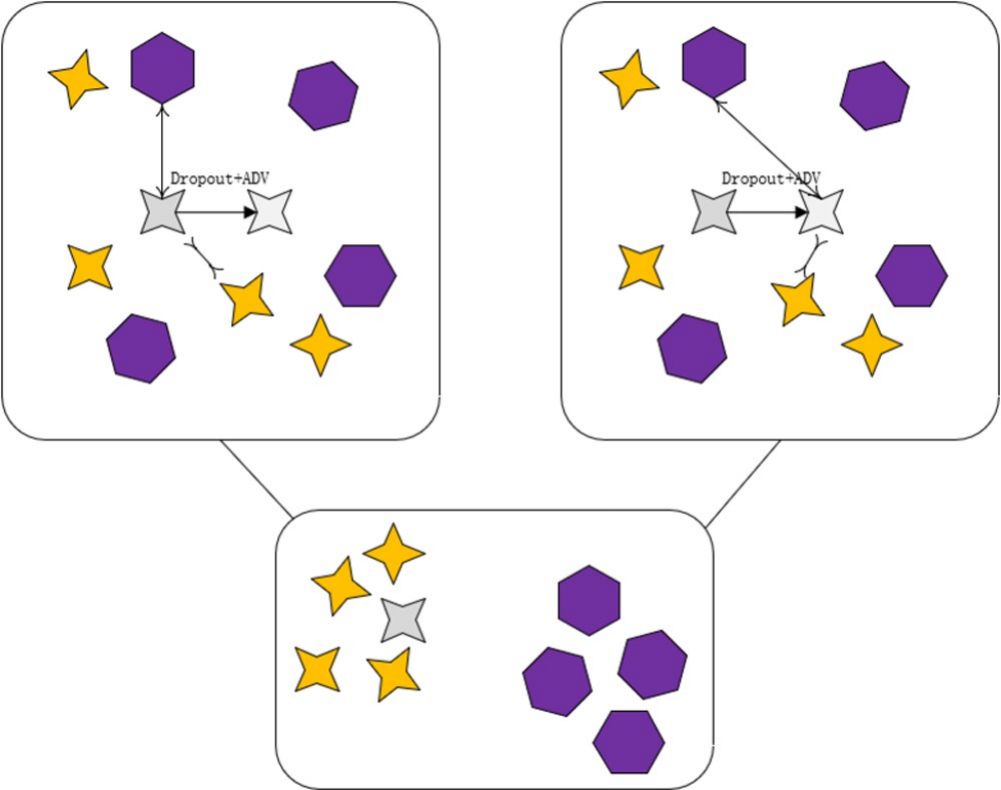
Double comparison learning.

We have built a new double comparison learning module as shown in Fig 4 by combining the more popular comparison learning nowadays. With the feature representation *Z*_*i*_ and the classifier *θ*_*i*_ of the input example *x*_*i*_, we try to align the softmax transform of $\theta_{i}^{T}z_{i}$ with the label of *x*_*i*_. $\theta_{i}^{*}$ denotes the column of *θ*_*i*_, corresponding to the *x*_*i*_ real label. We expect the dot product $\theta_{i}^{*T}z_{i}$ to be the largest. Therefore, we turn to learning to better represent *θ*_*i*_ and *z*_*i*_ with supervised signals. Here we define the dual contrastive loss to exploit the relation between different training samples, which tries to maximize $\theta_{i}^{*T}z_{j}$ if *x*_*j*_ has same label with xi while minimizing $\theta_{i}^{*T}z_{j}$ if *x*_*j*_ carries a different label with *x*_*i*_.

Given an anchor *z*_*i*_ originating from the input example *x*_*i*_, we take ${\left\{\theta_{j}^{*}\right\}}_{j\in P_{i}}$ as positive samples and ${\left\{\theta_{j}^{*}\right\}}_{j\in A_{i}\setminus P_{i}}$ as negative samples and define the following contrastive loss:

$$L_{z}=\frac{1}{N}\sum_{i\in I}\frac{1}{\left|P_{i}\right|}\sum_{p\in P_{i}}-log\frac{\exp\left(\theta_{i}^{*}\cdot \frac{Z_{i}}{\tau }\right)}{\sum_{a\in A_{i}}\exp\left(\theta_{a}^{*}\cdot \frac{Z_{i}}{\tau }\right)}$$ (9)

where *τ* ∈ *R*^+^ is the temperature factor, $A_{i}:=I\{i\}$ is the set of indexes of the contrastive samples and $P_{i}:=\left\{p\in A_{i}:y_{p}=y_{i}\right\}$ is the set of indexes of positive samples, and |*P*_*i*_| is the cardinality of *P*_*i*_.

Similarly, given an anchor $\theta_{i}^{*}$, we can also take ${\left\{Z\right\}}_{j\in P_{i}}$ as positive samples and ${\left\{Z\right\}}_{j\in A_{i}\backslash P_{i}}${*Z*_*j*_}_*j*∈*Ai*\*P*_*i*__ as negative samples and define another contrastive loss:

$$L_{\theta }=\frac{1}{N}\sum_{i\in I}\frac{1}{\left|P_{i}\right|}\sum_{p\in P_{i}}-log\frac{\exp\left(\theta_{i}^{*}\cdot \frac{Z_{p}}{\tau }\right)}{\sum_{a\in A_{i}}\exp\left(\theta_{i}^{*}\cdot \frac{Z_{a}}{\tau }\right)}$$ (10)

The two-channel loss function is a combination of losses for two different negative samples:

$$L_{D}=L_{z}+L_{\theta }$$ (11)

## 4 Experiments

In this section, we conduct experiments such as model comparison tests, ablation experiments, and visualization on the publicly available Twitter15 [48], Twitter16 [48], and PHEME [49] datasets to illustrate the effectiveness of our model.

### 4.1 Datasets

We evaluated the AGAD model on three publicly available true datasets: Twitter15, Twitter16, and PHEME, all of which were collected from Twitter, the most influential social media site in the United States. Both Twitter15 and Twitter16 contain four targets: Non-rumor (N), False Rumor (F), True Rumor (T), and Unverified Rumor (U), for four levels of classification. PHEME includes two types of tags: rumor (R) and non-rumor (N) for rumor and non-rumor dichotomization. Moreover, a graph-based representation of posts is created by utilizing user, source, and comment information from the aforementioned datasets. Each node in the graph is encoded with BERT to represent the textual content, while the graph structure is encoded using GAT. Detailed statistics are shown in Table 1.

**Table 1:** Statistics of the datasets.

Statistic	Twitter15	Twitter16	PHEME
# of users	276663	173487	48843
# of posts	331612	204820	197852
# of tree	1490	818	6425
# of rumors	374	205	4023
# of false-rumors	370	205	2402
# of unverified rumors	374	205	
# of non-rumor	372	205	
Avg text length	15.84	15.87	17.79
Max text length	136	383	78
Min text length	2	2	2

### 4.2 Experimental settings

We make comparisons with the following state-of-the-art baselines:

SVM-TS [3] is a linear SVM classifier that can use handcrafted features to capture the variation of social context features.

CNN [6] is a CNN-based model that can learn the local spatial features between rumor posts.

BERT [46] is a popular pre-trained model that is used for rumor detection.

RvNN [50] is based on bottom-up and top-down tree recursive neural models for learning and classification of rumor representations, and they conform to the propagation layout of tweets.

GCAN [5] uses a dual attention mechanism based on GCN to interpret structural information about text, user features and propagation paths.

UDGCN [7] directly uses GCN for rumor detection, in which the root feature enhancement strategy is used to improve the performance of the model.

BiGCN [7] uses two GCNs based on source-posted information, one to learn the propagation pattern and one to learn the scattering pattern.

GACL [45] is a GCN-based model using adversarial and contrastive learning, which can not only encode the global propagation structure, but also resist noise and adversarial samples, and capture invariant features.

AGAD (our) is a dual contrast learning model based on GAT and including an attention mechanism with filters plus adversarial interference, which uses the properties of the attention mechanism to understand the propagation structure information more comprehensively, while also learning stronger invariant features through stronger adversarial samples.

The AGAD model proposed in this paper has a PyTorch implementation. I used a 3090 GPU for the experiments, python 3.9.16, and pytorch 2.0.1 to build the experimental environment. We randomly split the data into 5 parts and constructed 5 cross-validations. In addition, the learning rate was initialized to 5e-4 and gradually decreased during the training process based on a decay rate of 1e-4.

### 4.3 Results and discussion

Table 2 show the performance of all compared methods on the two public true datasets, with the bolded part performing the best and our model significantly outperforming all baselines. It is confirmed that there are advantages in the supervised rumor detection task in the attention graph model and in the dual ratio learning.

**Table 2:** Rumor detection results.

Twitter15						Twitter16					PHEME				
Method	Acc.	N	F	T	U	Acc.	N	F	T	U	Class	Acc.	Prec.	Rec.	F1
		F1	F1	F1	F1		F1	F1	F1	F1					
SVN-TS	0.642	0.811	0.434	0.639	0.6	0.691	0.763	0.483	0.722	0.69	R N	0.685	0.553 0.758	0.539 0.758	0.539 0.757
CNN	0.718	0.807	0.601	0.635	0.73	0.7	0.688	0.666	0.81	0.615	R N	0.747	0.683 0.768	0.512 0.872	0.584 0.816
RvNN	0.723	0.682	0.758	0.821	0.654	0.737	0.662	0.743	0.835	0.708	R N	0.763	0.689 0.796	0.587 0.858	0.631 0.825
BERT	0.735	0.731	0.722	0.73	0.705	0.804	0.777	0.525	0.824	0.787	R N	0.807	0.736 0.842	0.695 0.866	0.713 0.853
GCAN	0.842	0.844	0.846	0.889	0.8	0.871	0.857	0.688	0.929	0.901	R N	0.834	0.769 0.871	0.758 0.874	0.761 0.872
UDGCN	0.834	0.827	0.866	0.885	0.756	0.867	0.789	0.846	0.903	0.878	R N	0.805	0.752 0.831	0.673 0.875	0.708 0.852
BiGCN	0.886	0.891	0.86	0.93	0.864	0.88	0.847	0.869	0.937	0.865	R N	0.824	0.753 0.861	0.734 0.872	0.741 0.865
GACL	0.901	**0.958**	0.851	0.903	0.876	0.92	**0.934**	0.869	0.959	0.907	R N	0.85	0.801 **0.871**	0.75 **0.901**	0.772 0.855
AGAD	**0.935**	0.893	**0.984**	**0.947**	**0.913**	**0.95**	0.926	**0.952**	**0.993**	**0.915**	R N	**0.882**	**0.906** 0.835	**0.911** 0.830	**0.908** 0.831

Machine learning SVM-ST performs relatively poorly among all models, and the early deep learning models CNN and RvNN test results have improved relative to machine learning but perform relatively average in the new models of the moment. CNN only acquires features with local information, while RvNN can learn the structural information of rumor tree from top-down and bottom-up, which makes it obtain better performance than CNN.

BERT is a product of the nature of change in NLP models, which can generate better article representations and help the model understand the semantics to have more accurate control over feature extraction, which helps to improve the accuracy of article prediction.

GCAN, UDGCN, BiGCN and GACL are all GCN-based models, which are currently the first benchmarks for comparison with our model. The above four graph neural network models have been greatly improved relative to the previous deep learning models. Among them, GCAN mines the relationship between rumor propagation structure, user characteristics and contextual information using a dual co-attentive mechanism. UDGCN and BiGCN capture the relationship between global structural features, user features and contextual information of rumor trees by virtue of the GCN encoder. However, BiGCN fuses rumor bottom-up and top-down structural information to obtain better performance. GACL is an upgrade from GCN, which incorporates contrast learning and adversarial training to improve by 2%-4% compared to the previous one. The AGAD proposed in this paper outperforms all benchmark tests and improves accuracy by 4.1%, 3% compared to GACL when tested on the four-classified Twitter15 and Twitter16. The improvement is 3.2% on the dichotomous PHEME dataset.

**Fig 5:**
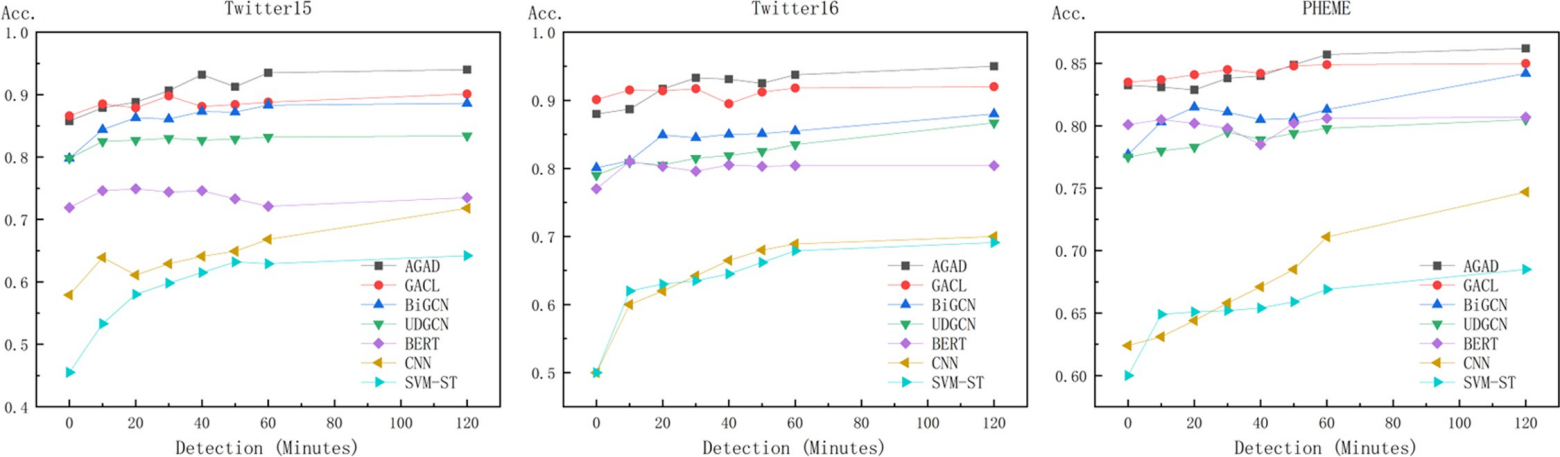
Early detection results of rumors for each dataset.

Early detection of rumors is a critical aspect in assessing the effectiveness of models designed for rumor detection. The objective of early detection is to identify rumors at an early stage before they propagate widely and subsequently lead to notable social consequences. As shown in Fig 5, the study presented herein employs a time frame of eight specific moments, i.e., 10,20, and 120 minutes, for evaluating the capability of the model to accurately detect rumors based on the limited initial information available.

Fig 5 shows the performance of our AGAD model and other different benchmarks in the early rumor detection task. It can be observed that in the early stage, the performance is not well released due to the lack of training data. In the second half the models all enter a smooth phase. The graph neural network based models have better accuracy compared to other types of models. Our AGAD models all eventually beat the other benchmarks, proving the feasibility of attention filters, adversarial training and double comparison learning.

This was made possible by the following theoretical foundations:

1. A filter with an attention mechanism is used, which acts as a good filter for noise magazines. Since the graph neural network itself has no mechanism for filtering, it also takes noise as a learning object, which eventually affects the performance of the model.
2. The Graph Attention Network (GAT) is utilized to encode the inherent topological characteristics among rumor posts. Meanwhile, the Bidirectional Encoder Representations from Transformers (BERT) model is applied to encode the textual information of each graph node. BERT is an advanced pre-trained model, capable of dynamically adjusting word embeddings based on the context, to tackle the challenge of multiple meanings. Also GAT utilizes an attention mechanism to make the weight relationship between each post construct more realistic structural information.
3. Adversarial training is used, and in the real world, there are some artificially perturbed samples in addition to noisy samples. Obviously, ordinary data augmentation cannot handle such adversarial samples. There are many means of camouflage, generative and perturbative, and perturbation is the effect of camouflaging in a high-dimensional space so as to evade the detection model. The adversarial features generated by the adversarial module based on adversarial training can effectively simulate this phenomenon. We designed a perturbation-based adversarial training module. Also this adversarial training assists us to add down the contrast learning.
4. Double contrast learning is used. We propose double contrast learning in order to learn more angular features, and also use the more popular dropout generation such as samples which is well matched with our adversarial training module, so that our negative samples get stronger adversarial performance, allowing our model to learn more angular effective features, and the practice also proves our idea.

### 4.4 Ablation study

To prove the efficiency of the different modules of AGAD, where AGAD_NAF denotes the model with the attention filter removed; AGAD_NDCL denotes the dual comparison learning removed; and AGAD_NADV denotes the adversarial training removed. We compare them with the following variants.

#### 4.4.1 The effect of attention filters on the model

**Fig 6:**
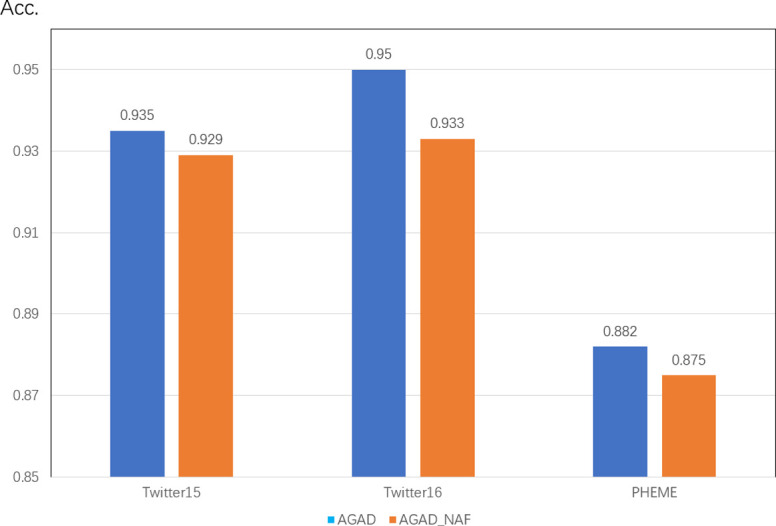
Performance of the attention filter on three public datasets.

AGAD_NAF removes the attention filtering module as shown in Fig 6, and the accuracy rates on Twitter15, Twitter16 and PHEME datasets each have different degrees of decrease, with Twitter16 dataset decreasing by 1.7 percentage points and the other two datasets both decreasing by nearly 1 percentage point. The filter based on the attention mechanism proved to be effective in filtering the invalid or negative noise of comments in rumor threads, enhancing the robustness of the model.

#### 4.4.2 The effect of adversarial training on the model

**Fig 7:**
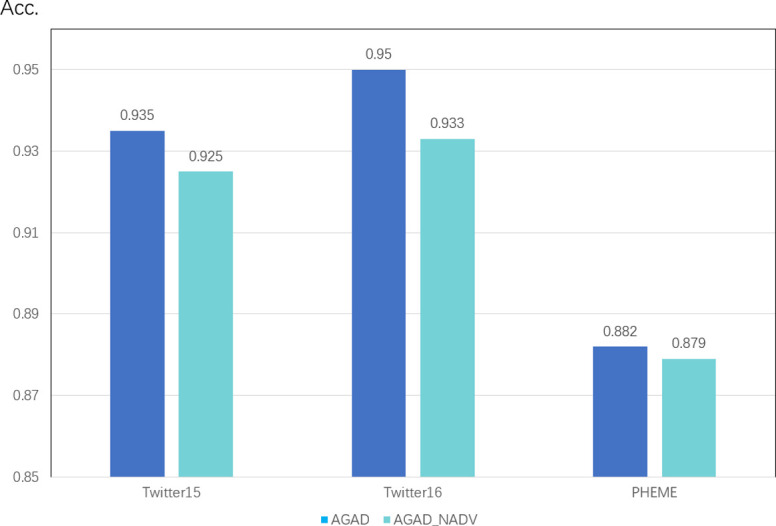
Performance of adversarial training on three public datasets.

GAD_NADV removes the adversarial training module as shown in Fig 7, and the accuracy drops more than 1 percentage point on both the Twitter15 and Twitter16 datasets, and the adversarial training module both play a good effect, but in the PHEME dataset there is an improvement but not a particularly large improvement, probably because the PHEME dataset has a relatively large amount of data, and finally the adversarial training did not achieve particularly good results. However, from the perspective of the final average performance of the three datasets. The adversarial training’s played a good effect in simulating the malicious hostile comment environment.

#### 4.4.3 The effect of double comparison learning on the model

**Fig 8:**
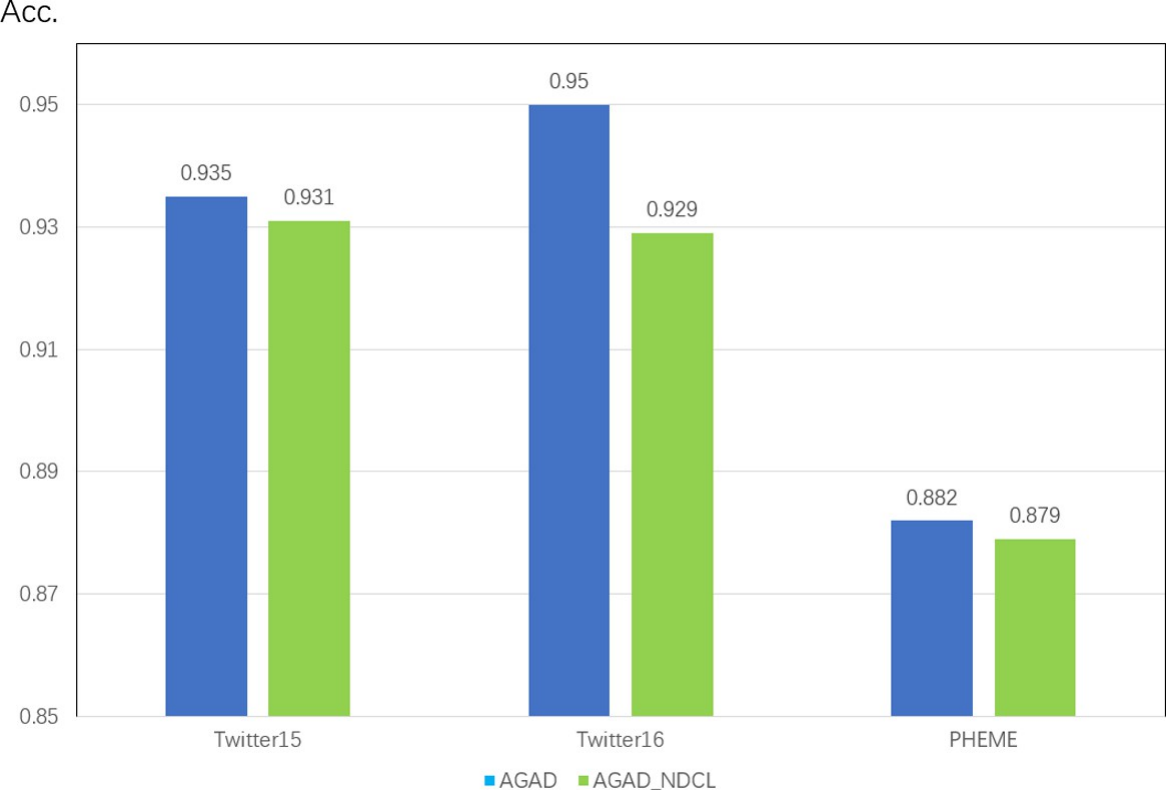
Performance of double comparison learning on three public datasets.

AGAD_NDCL removes the double comparison learning module as shown in Fig 8, and the accuracy drops by 2.1 percentage points on the Twitter16 dataset, which is very effective. In the Twitter15 and PHEME datasets there is a slight decrease but the effect is not significant. Obviously, the effect of double contrast learning is still a relatively significant performance improvement after learning the features of source and comment nodes in conversation threads through different perspectives and fusing them.

#### 4.4.4 Current graph contrastive learning

We use contrastive learning thanks to the excellent performance of contrastive learning in the field of graphics in recent years. But there were some difficulties involved. Contrast learning is not particularly widely used in the text domain, although we took rumors and constructed nodes and produced graphs through conversation threads. But this graph structure is not the same thing as a picture. We have also done some experiments on whether contrast learning can be used effectively. In the original baseline model, contrast learning was used but the performance was very bad, and there was a serious negative growth when scaling up the contrast learning usage. In order to solve this problem we optimized negative samples and also optimized contrast learning and finally constructed a double contrast learning that is more effective for text.

**Fig 9:**
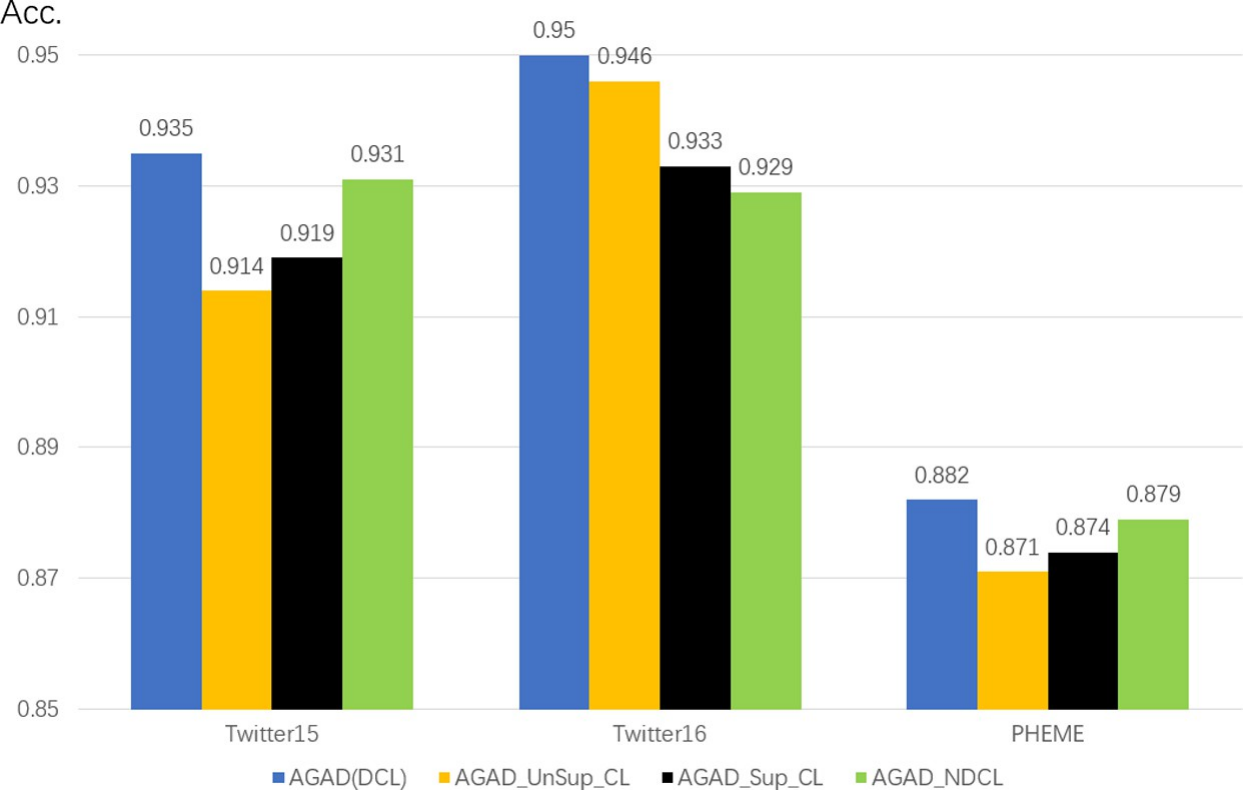
Performance of double contrast learning, self-supervised contrast learning and no contrast learning.

We worked through the work of Chu et al, Luo et al, and Ju et al. [51–53] on graph contrastive learning methods and we did the following experiments. Fig 9 shows the performance of our proposed double contrast learning and self-supervised contrast learning. It is easy to see that our dual contrast learning achieves the best results. However, traditional self-supervised contrast learning with optimized samples only improves the performance of the model in Twitter16 over the model without contrast learning, and shows negative growth in both Twitter15 and PHEME datasets. Although traditional self-supervised contrast learning is not ideal in learning text features, there is still much room for exploration. Our dual contrast learning demonstrates this.

#### 4.4.5 Performance of different GNN architectures

**Fig 10:**
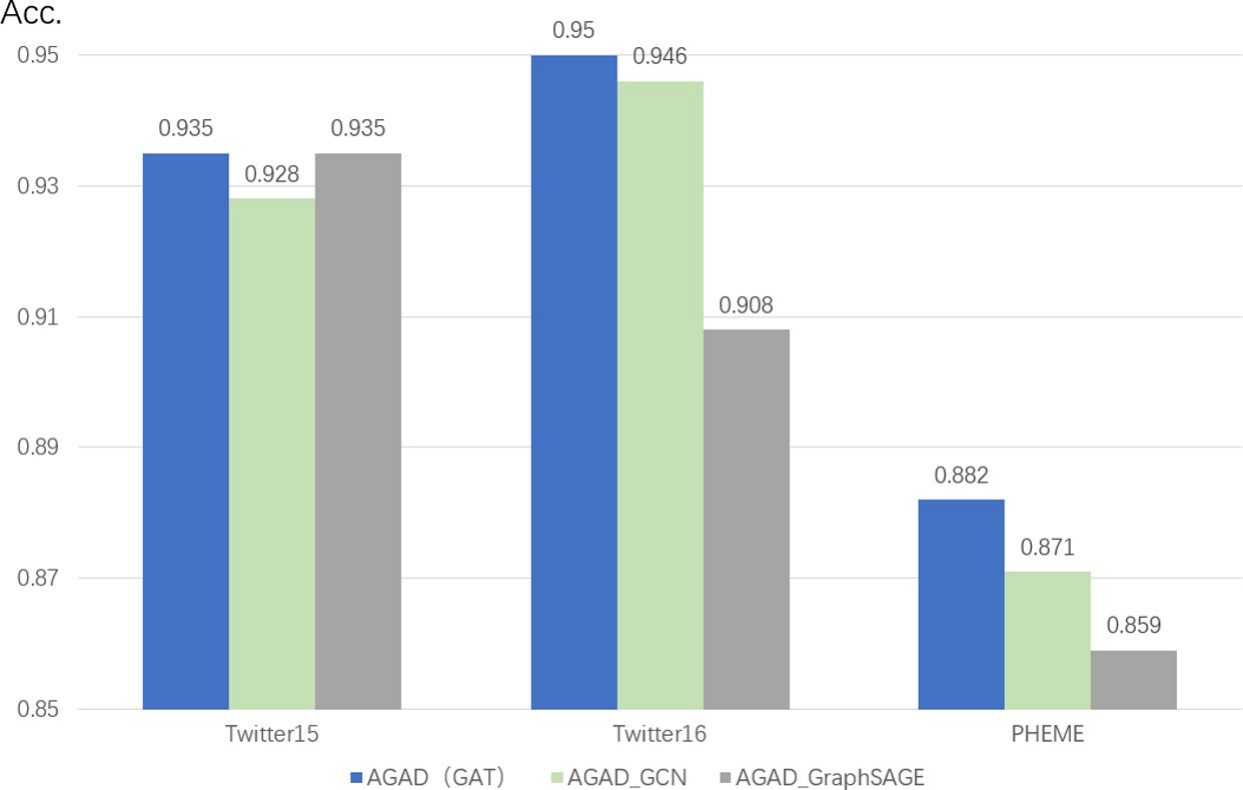
Performance of GAT, GCN and GraphSAGE architectures.

It is easy to see through Fig 10 the different performances of our model when choosing GAT, GCN and GraphSAGE. The results show that the GAT architecture has a clear advantage and it achieves good performance. It is not difficult to see that the GraphSAGE architecture reached a level of parity with GAT at one point in the Twitter15 dataset, but it performed poorly in the other two datasets. GraphSAGE is theoretically superior to GCN, but it does not perform as well as GCN in both the Twitter16 and PHEME datasets. From the output of papers in recent years we can easily see that scholars prefer to use GCN as the network underlying the model. Our experiments also verified this. So we finally chose GAT.

## 5 Conclusion

This paper presents a novel rumor detection model AGAD, proposed to address the challenge of identifying and analyzing rumors in social media. The framework includes attention mechanism filter, adversarial training and double contrast learning. The comprehensive evaluation and ablation study results show the effectiveness of the proposed rumor detector on three public datasets.

The pre-training phase uses BERT to obtain a vector representation of each post in AGAD. Then each post is noisy by attention mechanism filter, and the model is implemented to learn the high weight features by weighting out the low value features. Next, the robustness of the posts is reinforced by adding perturbation and interference to the filtered posts through adversarial training. Finally, the structural information of rumor propagation of conversation threads is encoded using GAT network with double comparison learning to capture more features of posts. The comprehensive evaluation and ablation study results show the effectiveness of the proposed rumor detector on three public datasets. In the future, we plan to work on multimodal information fusion and extraction for rumor detection.
